# Acceptability of the eHealth Intervention Sustainable Worker Digital Support for Persons With Chronic Pain and Their Employers (SWEPPE): Questionnaire and Interview Study

**DOI:** 10.2196/46878

**Published:** 2023-09-28

**Authors:** Frida Svanholm, Christina Turesson, Monika Löfgren, Mathilda Björk

**Affiliations:** 1 Pain and Rehabilitation Centre Department of Health, Medicine and Caring Sciences Linköping University Linköping Sweden; 2 Division of Prevention, Rehabilitation and Community Medicine Department of Health, Medicine and Caring Sciences Linköping University Linköping Sweden; 3 Department of Clinical Sciences Karolinska Institutet Stockholm Sweden; 4 Department of Rehabilitation Medicine Danderyd Hospital Stockholm Sweden

**Keywords:** chronic pain, digital support, eHealth, return to work, rehabilitation, support, quality of life, implementation, acceptability, interview, questionnaire, qualitative, barrier, users, mobile phone

## Abstract

**Background:**

Sick leave and decreased ability to work are the consequences of chronic pain. Interdisciplinary pain rehabilitation programs (IPRPs) aim to improve health-related quality of life and participation in work activities, although implementing rehabilitation strategies at work after IPRPs can be difficult. Employers’ knowledge about pain and the role of rehabilitation needs to be strengthened. The self-management of chronic pain can be improved through eHealth interventions. However, these interventions do not involve communicating with employers to improve work participation. To address this deficiency, a new eHealth intervention, Sustainable Worker Digital Support for Persons with Chronic Pain and Their Employers (SWEPPE), was developed.

**Objective:**

This study aimed to describe the acceptability of SWEPPE after IPRPs from the perspective of patients with chronic pain and their employers.

**Methods:**

This study included 11 patients and 4 employers who were recruited to test SWEPPE in daily life for 3 months after IPRPs. Data were collected using individual interviews at the end of the 3-month test period and questionnaires, which were completed when SWEPPE was introduced (questionnaire 1) and at a 3-month follow-up (questionnaire 2). Data were also collected on how often SWEPPE was used. Qualitative data were analyzed through a qualitative content analysis using an abductive approach. The framework used for the deductive approach was the theoretical framework of acceptability. Quantitative data were analyzed through descriptive statistics and the differences between the responses to questionnaires 1 and questionnaire 2 using the Wilcoxon signed rank test.

**Results:**

Both patients and employers reported that SWEPPE increased their knowledge and understanding of how to improve work participation and helped them identify goals, barriers, and strategies for return to work. In addition, participants noted that SWEPPE improved employer-employee communication and collaboration. However, experiences and ratings varied among participants and the different SWEPPE modules. The acceptability of SWEPPE was lower in patients who experienced significant pain and fatigue. A high degree of flexibility and choice of ratings in SWEPPE were generally described as helpful.

**Conclusions:**

This study shows promising results on the user acceptability of SWEPPE from both patient and employer perspectives. However, the variations among patients and modules indicate a need for further testing and research to refine the content and identify the group of patients who will best benefit from SWEPPE.

## Introduction

### Background

The use of information and communication technology to enable or improve health care, that is, eHealth, is constantly growing around the world. The advantages of eHealth include ease of use (ie, the self-management of health), ease of access, and reduced health care costs. However, to increase the quality of eHealth solutions and make them more accessible to the people who need them the most, further research and development are necessary [1]. One field in which eHealth solutions are used is chronic pain prevention and treatment [2]. Many people experience chronic pain (pain lasting >3 months). In Europe, approximately 20% of the population experiences moderate to severe pain [3]. Chronic pain often results in sleep disturbances, increased stress, decreased mental health, and decreased overall quality of life, conditions that negatively affect everyday activities, social life, and work [4]. Effective interventions are needed to help people manage their pain as well as its secondary effects [5]. Different eHealth solutions, including mobile apps for the self-management of pain, complement traditional health care by reducing pain intensity and improving disabilities [2,6-8]. Patients who experience chronic pain have expressed a need for self-management through eHealth to obtain information and knowledge about pain and management strategies, help them accomplish everyday tasks, and improve communication and social participation [9]. In addition, eHealth can help patients with chronic pain improve their motivation, support their goal setting, provide a place for feedback, and support them after rehabilitation when professional support is no longer present [10].

Patients with chronic pain often report decreased work ability and increased absence from work [11,12]. Interdisciplinary pain rehabilitation programs (IPRPs) aim to support people with chronic pain to improve their function, performance of activities, and quality of life. IPRPs also aim to reduce sick leave and improve return to work (RTW). IPRPs include education, physical training, cognitive behavioral therapy, and a social or work component [13,14]. IPRPs in the Swedish context have shown promising results concerning RTW from a 2-year follow-up perspective [15]. However, patients participating in IPRPs in Sweden have expressed a need for improved support for RTW [16]. Furthermore, Swedish employers have described economic challenges prioritizing RTW support. In the context of business pressure, the ability and willingness of employers to take social responsibility for sick-listed workers can be affected. For example, the nature of a specific job and the value of a specific employee might guide the priority [17]. Recently, legislation in Sweden regarding employers’ role and responsibilities in the RTW process has been strengthened. For example, recent legislation requires employers to devise a plan for RTW, including work-related goals and adaptations of work tasks [18]. Both patients and other stakeholders involved in the RTW process for patients with chronic pain have described the importance of employers’ support for RTW. However, employers’ knowledge of chronic pain, rehabilitation, and work adaptations needs to be strengthened for them to fulfill their responsibilities [16,19]. Clearly, regular communication and an employer’s understanding, including adjustments at the workplace, can facilitate RTW [20-23].

Although there is a growing set of eHealth solutions for patients with chronic pain supporting self-management, none of the solutions include the work situation or focus on support for RTW. To strengthen the role of the employer in the RTW process for cancer survivors, a web-based intervention was developed [24]. However, to the best of our knowledge, patients with chronic pain and their employers have no similar support systems in place.

To improve support for patients with chronic pain and their employers in the RTW process after IPRPs, an eHealth intervention was developed. The Sustainable Worker Digital Support for Persons with Chronic Pain and Their Employers (SWEPPE) intervention consists of a smartphone app for patients and a web application for employers. The smartphone app includes the following 6 modules: an action plan, daily self-rating, self-monitoring graphs, a coach, a library, and shared information with the employer. The web application includes the following 2 modules: the library and shared information with the employer [25]. SWEPPE was developed stepwise by a multidisciplinary research team that included health care researchers, a user representative, and a software team. Reference groups representing the end users (ie, patients with chronic pain and their employers) participated in the different stages of the development process. They provided information regarding the desired features and content in SWEPPE, participated in usability tests, and provided feedback on the functions in SWEPPE. The development study showed that SWEPPE was perceived as a useful tool with an appealing interface and safe, logical, and relevant characteristics that motivated further use and testing [25]. Feasibility studies evaluate the quality of an intervention before moving on to more large-scale studies [26]. Acceptability, an important aspect of feasibility studies, concerns the appropriateness and usefulness of an intervention as perceived by the intended users [27-30]. Sekhon et al [30,31] defined acceptability as “a multi-faceted construct that reflects the extent to which people delivering or receiving a healthcare intervention consider it to be appropriate, based on anticipated or experiential cognitive and emotional responses to the intervention,” and identified a distinction between prospective (preintervention) and retrospective (postintervention) acceptability.

### Objective

This study aimed to describe the acceptability of SWEPPE after IPRPs from the perspective of patients with chronic pain and their employers.

## Methods

### Study Design

To describe the acceptability of the eHealth intervention SWEPPE, a combination of qualitative and quantitative longitudinal data was used. The theoretical framework of acceptability (TFA), developed by Sekhon et al [30], was used in the analysis process.

### Participants and Recruitment Process

This study is part of the feasibility testing of SWEPPE after IPRPs. Patients who had participated in IPRPs both within primary and specialist care in Region Östergötland, Sweden, were recruited to test SWEPPE for 3 months. IPRP staff identified patients eligible for participation. If patients expressed interest in the study, they provided the IPRP staff with their contact details. This information was sent by email to the first author (FS), who contacted the patients and provided them with both written and oral information about the study. If patients consented to participate, they were asked to invite their employers to participate in the study. During the test period, the participants were encouraged to use SWEPPE in their daily life. At the end of the test period, all participants were invited to a follow-up interview. The inclusion criteria for this study were as follows: individuals aged 18 to 65 years who completed IPRPs and were on sick leave or had returned to work after IPRPs; eligible participants took part in the test for 3 months and in a follow-up interview. In total, 11 patients and 4 employers participated in this study.

### Start-Up Process

An individual digital introduction meeting, via Skype (Skype Technologies) or Zoom (Zoom Video Communications, Inc), was scheduled at the start of the test period for each patient and employer separately. Before this introduction meeting, each patient was sent a log-in code to SWEPPE. At the meeting, the different modules of SWEPPE were introduced and an action plan was developed, which focused on work-related goals, barriers, strategies, and support needed from the employer. SWEPPE was introduced by an occupational therapist, that is, the first author (FS), who was familiar with SWEPPE and had clinical experience with IPRPs. The focus of the meeting was on the modules and functions in SWEPPE rather than professional support in the choices of, for example, goals and strategies. Both the patient and employer were informed that for the employer to access SWEPPE, the patient had to actively share information with their employer in their app. Participants were encouraged to contact the research team if they had questions regarding the use and function of SWEPPE. No further meetings were scheduled until the follow-up after 3 months.

### The SWEPPE Intervention and Study Context

**Figure 1:**
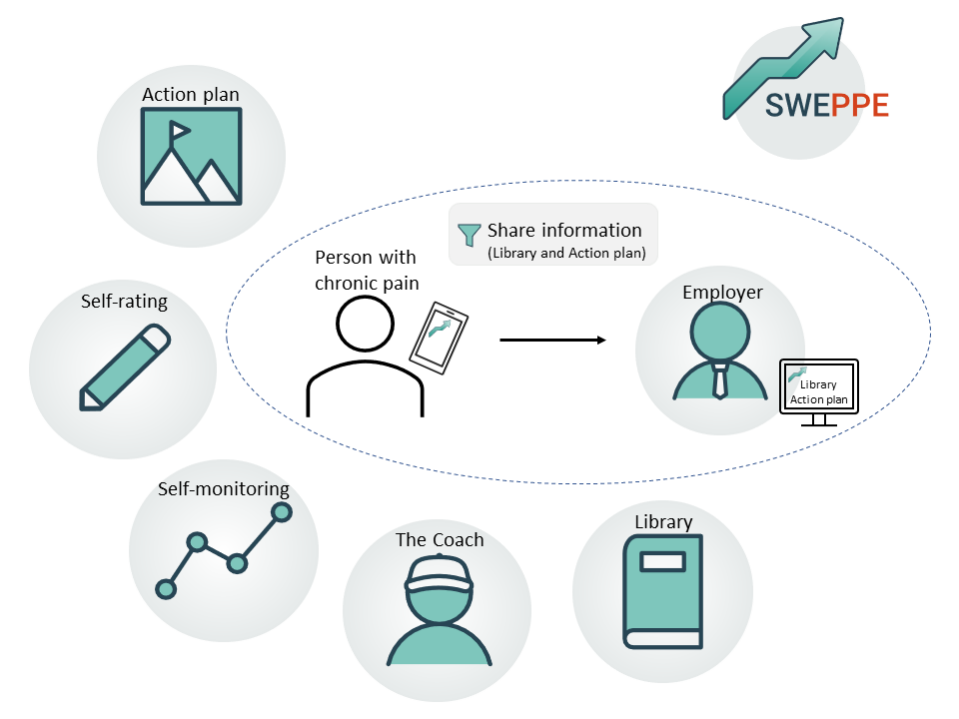
The 6 modules in Sustainable Worker Digital Support for Persons with Chronic Pain and Their Employers (SWEPPE).

SWEPPE is an eHealth intervention containing 6 modules in the SWEPPE mobile phone app and 2 modules in the SWEPPE web application. For example, the action plan involves goal setting; the identification of barriers, strategies, and support needed from the employer; the daily self-rating of health and activity variables; and self-monitoring graphs concerning both weekly follow-up of the work-related goals and daily self-rating variables [25]. Figure 1 provides an overview of the modules, and Table 1 provides a description of the content of each module. SWEPPE is intended to be self-administered. Except for the coach module, no professional support was included in the intervention. Each participant decided on what modules and functions to use and how to use them. SWEPPE was tested in the context of IPRPs, that is, after the rehabilitation programs were completed. The IPRPs in this study were group-based intervention programs lasting between 6 and 10 weeks within primary and specialist care in Region Östergötland, Sweden. During the IPRPs, patients worked with individual goals and strategies to improve their health and participation in activities and work. Professions involved in the IPRPs could be physical therapists, occupational therapists, psychologists, and physicians.

**Table 1:** Description of Sustainable Worker Digital Support for Persons with Chronic Pain and Their Employers (SWEPPE) modules and their functions.

Module in SWEPPE	Functions
The action plan^a^	Goal setting in relation to work; the identification of barriers to RTW, strategies to handle the barriers, and support needed from the employer; and weekly evaluation of work ability and the fulfillment of the goals
Daily self-rating^a^	Self-rating of health and psychosocial aspects, work situation, and strategies
Self-monitoring graphs^a^	Graphs for self-monitoring health and psychosocial aspects, work ability, and progress toward the goal over time
The coach^a^	Opportunity to ask a question and receive a written answer from a coach
The library^a,b^	Knowledge database developed based on previous research with information (texts, films, and audio clips) that reflects a biopsychosocial perspective of chronic pain, physical activity, managing the situation, activity pacing, balance in daily life, sleep, and workplace adaptations; tools for dialogue; and answers from the coach to common questions
Shared information with the employer^a,b^	The person with chronic pain can give the employer access to the library and share information from the action plan and the graph for monitoring work ability and goal fulfillment in SWEPPE, and the employer receives the information from the parts of the action plan the employee has chosen to share; if the employee does not want to share any information from the action plan, the employer still has access to the library

^a^Modules included in mobile phone app for patients.

^b^Modules included in the web application for employers.

### Data Collection

#### Overview

The primary focus of this study was to describe user acceptability using qualitative data from interviews and free-text answers from questionnaires. As a complement, we collected quantitative data on the perceived support of SWEPPE from questionnaires and on patients’ use of SWEPPE during the test period from the app. This triangulation of data sources was used to ensure the credibility of the results.

#### Interviews

To collect data on the retrospective acceptability of SWEPPE, individual interviews on the experiences of using SWEPPE were conducted at the end of the 3-month test period. An interview guide with open-ended questions was used [32]. The interview guide consisted of a set of question areas. These areas included experiences of SWEPPE as a supportive tool (ie, the parts of SWEPPE identified as supportive and the parts that were missing or could be further developed), experiences of SWEPPE in the collaboration between the patient and employer, use of SWEPPE in the context of IPRPs, and the timing of SWEPPE. Follow-up questions were asked when needed to further understand or deepen the answers. The interview guide was used to ensure that no question areas were missed. Most interviews lasted for approximately 40 minutes.

Interviews were conducted by the first author either digitally (Zoom or Teams [Microsoft Corp]; 11 interviews) or via telephone (4 interviews) at the convenience of the participants. All interviews were digitally audio recorded and transcribed verbatim by a professional secretary.

#### Questionnaires

Data on patients’ and employers’ expectations (prospective acceptability) as well as experiences (retrospective acceptability) of using SWEPPE were collected through questionnaires. Two questionnaires were developed for patients and employers, respectively. The questionnaires included questions on personal characteristics and the same questions used in the development study [25] related to the modules and functions of SWEPPE. Questions were rated on a 0-to-100 visual analog scale, and the responders were given the possibility to add free-text answers. For example, questionnaire 1 for patients (Q1P) and questionnaire 1 for employers (Q1E) asked the respective participants to rate the support *they expected* from SWEPPE concerning identifying goals and developing work ability, and questionnaire 2 for patients (Q2P) and questionnaire 2 for employers (Q2E) asked the respective participants to rate the support *they received* from SWEPPE concerning identifying goals and developing work ability. The visual analog scale ranged from 0 (no support) to 100 (best possible support). The questionnaires were digital and sent to participants via email. Q1P and Q1E were sent to the respective participants after the introduction meeting, and Q2P and Q2E were sent to the respective participants before the follow-up interview. After 1 week, up to 2 reminders were sent to participants who did not return the questionnaires.

#### SWEPPE User Data

During the test period, data regarding patients’ use of the SWEPPE app were saved on a database. After the test period, data concerning the modules self-monitoring (number of weekly follow-up ratings), self-rating (number of daily scoring on any variable), action plan (number of registered employer support), and the coach (number of times the coach function was used) were extracted from the database to an Excel (Microsoft Corp) file.

### Analysis

#### Qualitative Analysis of Interviews and Free-Text Answers in Questionnaires

A combination of deductive and inductive qualitative content analyses, that is, an abductive approach, was used as described by Patton [32]. First, the interview data and the qualitative data from the questionnaires were analyzed using a deductive approach guided by the 7 components of acceptability (affective attitude, burden, ethicality, intervention coherence, opportunity costs, perceived effectiveness, and self-efficacy) proposed by Sekhon et al [30] in a TFA. The deductive approach structured the analysis around and focused the analysis on the acceptability concept [33,34] using the 7 components from the TFA as predetermined categories. The free-text answers in Q1P and Q1E were the base for the analysis of prospective acceptability, whereas both the interviews and the free-text answers from Q2P and Q2E were the base for the analysis of retrospective acceptability.

The qualitative analysis was performed in Microsoft Word (Microsoft Corp). In the deductive phase of the analysis, a table with the 7 TFA components was created. The table included 1 row for each TFA component. Next, each questionnaire and interview transcript (ie, each unit of analysis) were read thoroughly. Text units from the transcripts were copied and sorted into the appropriate row in the table, depending on what component of acceptability it concerned. Therefore, the TFA components formed categories in a theory-driven manner. When all texts were sorted into the table, each row (ie, component of acceptability) was further analyzed using a more inductive approach, grounded in the piece of text under each TFA component. Each TFA component is theoretically broad and described in general, which made it possible to inductively analyze each component. This approach openly defined the content of each category. In this phase, the text units were condensed, coded, and labeled using the participants’ own words as much as possible. Then, similar codes were sorted into subcategories [32]. The analyses of prospective and retrospective acceptability were initially performed separately. Finally, the prospective subcategories and retrospective subcategories were compared and condensed.

The first author (FS) performed the interviews and analyses. To ensure the credibility of the results, there were recurrent discussions among all the authors during data collection and analyses. Categories and subcategories were discussed until a consensus was reached. Two authors (MB and CT) were involved in the development of SWEPPE. One author (FS) was well versed in SWEPPE, and the fourth author (ML) did not have experience with SWEPPE before this study. The research group had clinical experience of work interventions and IPRPs (FS) as well as several years of experience in pain and rehabilitation research (MB, ML, and CT).

#### Questionnaires

Quantitative data from the questionnaires were extracted to SPSS Statistics (version 26; IBM Corp), where the differences between the responses to questionnaire 1 and questionnaire 2 for each question were analyzed using the Wilcoxon signed rank test. A critical *P* value of ≤.05 was used to determine statistical significance. Descriptive statistics were calculated for questionnaire 1 and questionnaire 2 separately and presented as median and IQR for each question. The numbers of patients and employers with positive and negative differences between questionnaire 1 and questionnaire 2 for each function were also analyzed.

#### SWEPPE User Data

Frequency of the use of each function was calculated.

### Ethical Considerations

All the participants in the study were provided written and oral information about the study. The participants were notified that their participation was voluntary and could be withdrawn at any time. All the patients and employers provided their written informed consent. Participants did not receive any compensation for participation in the study. Data were handled confidentially (eg, interviews and questionnaires were coded with specific ID numbers). Data were stored on highly secure databases. The Swedish Ethical Review Board Authority approved the study (Dnr 2020-01593).

## Results

### Participants

An overview of the patient characteristics and the participation of patients in different parts of the study is presented in Table 2. Overall, 11 patients and 4 employers participated in this study. Background variables were available for 9 (82%) of the 11 patients, as 2 (18%) patients did not complete questionnaire 1, where these data were collected. Moreover, 10 (91%) of the 11 patients and 3 (75%) of the 4 employers were women. The mean age of the patients was 42.5 (SD 5.2; median 43) years, and that of the employers was 48.8 (SD 7.1; median 49) years. A total of 3 (27%) of the 11 patients were on 50% sick leave, and the duration of sick leave ranged from 0 to 3 months to >24 months. Among the 11 patients, 7 (64%) worked in the municipality in caring or teaching occupations, 1 (9%) was an IT consultant, and 1 (9%) worked with the administration. Both the duration of employment at the current workplace and time spent with the same employer ranged from 0 to 6 months to >24 months (Table 2).

**Table 2:** Overview of patient characteristics and participation in parts of the study.

ID number	Age (years)	Gender	Sick leave^a^, %	Sick leave duration (months)	Type of work^b^	Time at workplace (months) ^c^	Time with employer (months)^d^	Questionnaires 1 and 2	Interview	SWEPPE^e^ data	Employer interview
1	46	Woman	50	>24	Teacher	>24	>24	✓	✓	✓	✓
2	37	Woman	0	4-6	IT consultant	13-24	13-24	✓	✓	✓	✓
3	43	Man	0	0-3	Student assistant	13-24	13-24	✓	✓	✓	✓
4	—^f^	Woman	—	—	—	—	—		✓	✓	
5	42	Woman	0	4-6	Curator	>24	7-12	✓	✓	✓	
6	—	Woman	—	—	—	—	—		✓	✓	
7	44	Woman	0	Preventive^g^	Teacher	0-6	0-6	✓	✓	✓	✓
8	52	Woman	0	Preventive	Nursery school nurse	>24	>24	✓	✓	✓	
9	36	Woman	0	Preventive	Support assistant	13-24	0-6	✓	✓	✓	
10	46	Woman	50	13-24	Administration	>24	>24	✓	✓	✓	
11	37	Woman	50	7-12	Teacher assistant	13-24	7-12	✓	✓	✓	

^a^Current sick leave at the time of filling questionnaire 1.

^b^Patients’ own description of the type of work.

^c^Duration of employment at the current workplace.

^d^Duration of employment with the same employer.

^e^SWEPPE: Sustainable Worker Digital Support for Persons with Chronic Pain and Their Employers.

^f^Not available.

^g^Preventive: sick leave to be able to participate in rehabilitation.

### Use of SWEPPE

Table 3 presents how the patients used SWEPPE, which varied among the patients and modules. During the test period, the participants performed self-rating of at least one variable for a median of 47 (range 9-90) days. The number of weekly follow-ups ranged from 0 to 12 (median 2). Among the 11 patients, the 4 (36%) patients whose employers participated in the interviews provided weekly follow-up ratings for 7 to 12 weeks, which was more frequent in relation to the other patients. In the action plan, the number of supports needed by patients from their employers ranged from 2 to 8; 3 (27%) of the 11 patients had added 1 or 2 supports at a time during the test period. The median number of wanted supports from employers was 3. Two patients used the coach function once during the test period.

**Table 3:** Data on the use of Sustainable Worker Digital Support for Persons with Chronic Pain and Their Employers (SWEPPE) for each participant.

ID	Self-monitoring: weekly follow-ups, n	Self-rating: days with any rating, n	Action plan: employer supports, n	Coach: times used, n
1	7	47	3	1
2	12	87	2	0
3	11	89	3	1
4	3	16	5	0
5	0	27	4	0
6	1	90	3	0
7	9	88	7^a^	0
8	1	90	2	0
9	2	75	3	0
10	0	25	7^a^	0
11	1	9	2^a^	0

^a^Patient added 1 to 2 supports during the test period.

### Acceptability

#### Overview

Table 4 presents the results on acceptability from questionnaires 1 and 2. Both the patients and employers exhibited great variations, and there were no significant differences at *P*≤.05 between prospective expectations and retrospective experiences of SWEPPE regarding any of the modules (Table 4). There was also a great variation in the ratings of each module, both prospectively and retrospectively. On the basis of this, it is likely that different participants appreciated different parts of SWEPPE. The variations in ratings, both among participants and among modules, were also mirrored in the results of the qualitative interviews, which are presented subsequently in the sections “Affective Attitude,” “Perceived Effectiveness,” “Intervention Coherence,” “Self-Efficacy,” “Burden,” “and Ethicality.”

The qualitative results of the interviews focused on acceptability are presented with categories based on the 7 TFA components of acceptability proposed by Sekhon et al [30] (Table 5).

**Table 4:** Perceived support of Sustainable Worker Digital Support for Persons with Chronic Pain and Their Employers (SWEPPE) based on questionnaires 1 and 2 and number of participants with a negative or positive difference between questionnaires 1 and 2.

		Questionnaire 1, median (IQR)	Questionnaire 2, median (IQR)	Difference between questionnaires 1 and 2, *P* value	Participants with a positive difference between questionnaires 1 and 2, n (%)	Participants with a negative difference between questionnaires 1 and 2, n (%)	Participants with missing data, n (%)
**VAS^a^ items rated (1-100) by patients (n=9)^b^**							
	Setting a work-related goal and following the progress	58 (50-75)	53 (38-69)	.87	4 (44)	3 (33)	2 (22)
	Identifying barriers to and strategies for RTW^c^	77 (54-83)	60 (21-91)	.26	3 (33)	6 (67)	0 (0)
	Self-monitoring health aspects and getting an overview	76 (71-95)	69 (31-90)	.18	3 (33)	4 (44)	2 (22)
	Sharing information with the employer	60 (37-81)	61 (23-87)	.61	3 (33)	4 (44)	2 (22)
	Asking questions and receiving answers from the coach	54 (47-68)	21 (0-81)	.99	2 (22)	2 (22)	5 (56)
	Using the library	75 (56-85)	58 (30-86)	.44	4 (44)	3 (33)	2 (22)
	Getting reminders for the daily self-rating of health aspects and weekly evaluation of goal fulfillment	83 (61-95)	85 (70-96)	.99	3 (33)	5 (56)	1 (11)
**VAS items rated (0-100) by employers (n=4)**							
	Information about the employee’s work-related goal	84 (73-87)	89 (67-94)	.72	3 (75)	1 (25)	0 (0)
	Information about barriers to RTW identified by the employee	86 (75-89)	90 (66-96)	.72	3 (75)	1 (25)	0 (0)
	Information about strategies identified by the employee	76 (58-85)	80 (61-92)	.47	3 (75)	1 (25)	0 (0)
	Information about support wanted from the employer	95 (82-98)	91 (68-95)	.07	0 (0)	4 (100)	0 (0)
	Follow the employee’s progress in a graph (weekly follow-up)	81 (57-92)	80 (44-97)	.99	2 (50)	2 (50)	0 (0)
	Using the library	72 (68-83)	75 (55-92)	.99	2 (50)	2 (50)	0 (0)
	To be reminded of using SWEPPE	90 (85-98)	44 (6-87)	.14	1 (25)	3 (75)	0 (0)

^a^VAS: visual analog scale.

^b^Of the 11 patients, 2 did not complete questionnaire 1.

^c^RTW: return to work.

**Table 5:** Categories and subcategories of acceptability.

Categories with TFA^a^ definition of each acceptability component	Subcategories generated inductively based on interview data and free-text answers in the questionnaires
Affective attitude: how an individual feels about the intervention	General feelings Design and function
Perceived effectiveness: the extent to which the intervention is perceived as likely to achieve its purpose	Knowledge and understanding Goals and strategies Collaboration between employee and employer Flexibility and precision Importance of the context
Intervention coherence: the extent to which the participant understands the intervention and how it works	Interpretation of graphs and components
Self-efficacy: the participants’ confidence that they can perform the behaviors required to participate in the intervention	General capabilities Remember to use SWEPPE^b^
Burden: the perceived amount of effort that is required to participate in the intervention	Time aspects Effort in relation to energy Technical issues
Ethicality: the extent to which the intervention has good fit with an individual’s value system	Privacy

^a^TFA: theoretical framework of acceptability.

^b^SWEPPE: Sustainable Worker Digital Support for Persons with Chronic Pain and Their Employers.

#### Affective Attitude

##### General Feelings

During the introduction to SWEPPE, both patients and employers expressed neutral as well as high expectations for SWEPPE. At the follow-up, overall positive feelings regarding SWEPPE were described, for example, *“*SWEPPE have been good, a really good concept” and “SWEPPE is good, very very good.” Some employers saw the potential of SWEPPE for people with conditions other than pain and not only in the context of IPRPs. One of the employers felt that she wanted to provide SWEPPE to all employees with health problems. However, one of the patients expressed that SWEPPE was not supportive at all.

##### Design and Function

The design and function of SWEPPE were important for the participants, as SWEPPE was perceived to be “living and interactive” and easy to comprehend and assimilate: “But I think it’s a nice little tool. Easy to understand, easy to manage and make to your own” (Employer 2). However, one of the employers described the contrast to be visually weak, which lowered their impression.

#### Perceived Effectiveness

##### Knowledge and Understanding

Both patients and employers retrospectively described that SWEPPE contributed to more knowledge and understanding about pain, its consequences, and the need for adaptations in work and everyday life. These contributions were also something the patients wished and hoped for at the time of the introduction. Some patients as well as employers perceived the library to be a good source of information with texts at just the right level and with a reasonable length. The patients believed that self-rating and self-monitoring helped them analyze their own health and behaviors. For example, understanding the relationships between different variables (eg, between pain and stress and between physical activity and sleep) contributed to the patients’ deeper understanding of their health patterns. This understanding made it easier to plan activities and strategies and to be kind to oneself.

For employers, the new level of knowledge provided insights into their employees’ prerequisites and needs. The patients described their employers as more familiar with the complexity of pain and the fact that the rehabilitation of chronic pain is a process: “I’m pretty sure that many employers think—well good, here came an intervention [IPRP] and then after IPRP they think you will work just fine—but this [SWEPPE] is a way to make the employer understand that it is a [long] process” (Patient 5).

Some patients wanted to share their daily ratings with their employer, as they thought that this could further deepen their understanding. For other patients, a good understanding of their situation by their employer at the start could explain why they did not experience any difference in understanding retrospectively.

##### Goals and Strategies

During the introduction, the patients and employers expressed a hope that SWEPPE would facilitate goal setting and be a source of strategies both practically and mentally. After the test period, the participants stated that the action plan could help define credible goals and strategies. The patients described that SWEPPE helped them keep track of rehabilitation. They also described a greater awareness of their needs and strategies. For example, based on their daily ratings and self-monitoring, some patients chose to prioritize physical and self-rewarding activities. Some were also more capable of making active choices regarding the use of time and medical consumption. Predefined work-related strategies in SWEPPE were experienced as relevant and applicable. Furthermore, the patients described that SWEPPE facilitated adaptations at work.

Of the 11 patients, the 2 (18%) who had used the coach function in SWEPPE believed that this module was very helpful. One of the patients found that SWEPPE helped them develop strategies and adaptations in relation to pain in everyday life and, therefore, provided support for acceptance: “When I have a lot of work to do and when I feel really, really tired and it feels as if my body will break in 1000 parts. Then SWEPPE is a lifeline. And maybe that sounds strange cause it’s just an app but some way it’s a very good thing because it makes me get structure on what I do and It makes me see that my strategies works ok” (Patient 7).

However, some disadvantages and suggestions for improvement were also reported. SWEPPE was described as helpful in identifying the consequences of pain (ie, being inactive when having more pain) but not in identifying what could lessen pain. It was also stated that SWEPPE visualized the relationships between the different variables in the graphs. If there were a longer period of negative relations and the trend was negative, it could be difficult for the participants to maintain their general mood and believe in the strategies. The participants suggested that SWEPPE could be improved by making it possible to plan activities using the self-rating and self-monitoring graphs, such as through a calendar that would enable more preventive actions rather than focus on follow-up.

##### Collaboration Between the Employee and Employer

Prospectively, the patients as well as employers expected SWEPPE to be supportive in the dialogue between the employee and employer. Employers expected clarity and comprehensibility about work rehabilitation as well as more insights into employees’ needs and expectations of adaptations at work, which could enable dialogue and collaboration. One of the patients thought that the quality of support from SWEPPE depended more on the basic relationship with the employer and the employer’s experiences with pain and rehabilitation than on SWEPPE itself.

At follow-up, it was described that SWEPPE contributed to a higher prioritization of rehabilitation activities by the employer. SWEPPE clarified the expectations on the employer concerning rehabilitation, and, with support from SWEPPE, the experience of some patients was that it was easier to ask for and implement adaptations at work. Some patients and employers emphasized the importance of SWEPPE’s connection to IPRPs and described a medical base as essential for its trustworthiness: “It is structured here, what I need and why. And also there is a connection to the library, information and research...and so it has been a help for me to actually ask for these things that it would have been hard for me to ask for otherwise [without SWEPPE]” (Patient 5).

The employers described SWEPPE as a valuable base for dialogue with their employee. It had been easier to be concrete, clear, and structured and focus on the most relevant queries. Thus, SWEPPE supported more effective talks, which made the follow-ups shorter and more frequent. Furthermore, it was perceived that SWEPPE could provide a more relaxed approach to work and RTW. However, some patients did not use SWEPPE in dialogue with their employer, either because of poor relations with their employer or because it had been a quite well-functioning period at work.

##### Flexibility and Precision

Retrospectively, the flexibility in SWEPPE was appreciated by the patients, including the possibility to write one’s own strategies, choose what variables are to be visible in the graphs, and make personal notes. At the same time, some patients expressed a wish for even higher flexibility and more options, that is, the possibility to choose their own variables to self-rate and representation of longer periods in the graphs. In other words, they wanted a more tailored or individualized approach.

SWEPPE was described as somewhat rough. For example, it was possible to rate hours of sleep each day but not the quality of sleep or the number of hours of continuous sleep. Some patients missed pain locations that were relevant to them for receiving the correct feedback, and it was not possible to rate each strategy separately. Therefore, they ignored these functions: “...but I have several strategies. I wanted one evaluation for each strategy, that I can see what different strategies I have. Because than I know that strategy was really good but that other was really bad today. I have not been able to use that. I chose not to use that” (Patient 1). One of the employers perceived the library to be too general and wished for more concrete examples. Neither the patients nor the employers prospectively described the need for flexibility and precision.

##### Importance of Context and Timing

Overall, the patients described a good relationship between SWEPPE and IPRPs. When IPRPs ended, it could be silent and scary. Then, SWEPPE gave a feeling of continuing support from health care, as it helped remind them about what was learned during IPRPs and about the strategies to continue the rehabilitation process: “And often, when you end a course, you manage to continue in two weeks or a month, and then you forget about doing these important things [strategies]. SWEPPE reminds you every day.... It’s an incredible tool to continue the rehabilitation on your own” (Patient 3). Furthermore, one of the patients was pleased that SWEPPE was developed at the department where she received her IPRP, as she had confidence in the people who worked there.

Some patients believed that SWEPPE could be the most valuable when returning to work or when trying to increase the amount of work. One of the patients thought that SWEPPE had the best effect when feeling worse because it provided support in analyzing the situation and a strategy for doing better. When the situation was stable, no variation was observed in the ratings. According to the patients, when goals are fulfilled and the collaboration with the employer works out, it may be time to stop using SWEPPE.

#### Intervention Coherence: Interpretation of Graphs and Functions

Prospectively, the patients were apprehensive about not understanding how ratings and graphs should be analyzed and interpreted. At follow-up, the patients were generally able to make these interpretations, which some patients thought was primarily due to IPRPs. During IPRPs, they learned about the biopsychosocial dimensions of pain and how to modulate their pain. According to some patients, this knowledge was necessary to use SWEPPE to its fullest potential. Without IPRPs, SWEPPE would have been more of a checklist than a tool for analysis and strategies. Despite the knowledge from IPRPs, some patients found it difficult to interpret the graphs and how the graphs could be used to improve their situation. One of the patients said that she did not receive much support from SWEPPE because it provided the same answers all the time, and she did not know how to use the information. Another patient expressed that she had gone astray and perhaps made her own (ie, wrong) conclusions without IPRPs. If SWEPPE is used without IPRPs, the patients wanted more descriptions of the functions, a thorough introduction, and someone to discuss the ratings and graphs with continually: “...it’s a bit tricky sometimes. Actually, I have an academic education and therefore some knowledge on how to interpret graphs. But some kind of support, maybe a person that can help, what to look for, what may be good to look for...” (Patient 2).

Some patients described misunderstanding some other functions, such as the weekly follow-up and the option to share information with employers. One of the employers did not understand the difference between goal fulfillment and satisfaction with goal fulfillment.

#### Self-Efficacy

##### General Capabilities

At the introduction, some patients expressed concern that the ratings would be given without reflection. In addition, they saw a risk of too much reflection when rating health variables and performing analysis every day. Furthermore, some patients were uncertain whether they had the ability to identify relevant goals and balance goal-focused work with recovery.

After the test period, one of the employers expected that goals would be set together with health care professionals because she did not believe in her or her employees’ capacity to do this by themselves. If goals are to be set by the employer and patient alone, there is a risk that the goals will not be specific enough to guide actions. Starting the action plan was experienced as an important part of SWEPPE that needed to be anchored to be trustworthy. Furthermore, the employers expressed the need for health care support in apprehending information from the library. One of the employers anticipated a risk of making too optimistic plans that result in failure: “If I build upon SWEPPE [in the rehabilitation plan], there has to be something solid behind, from those who know the rehabilitation paths in healthcare” (Employer 1).

Some patients did not use the library because it was difficult for them to read and assimilate text. They appreciated the films but could not fully use the library.

##### Remember to Use SWEPPE

During the introduction meeting, both patients and employers expressed that they did not trust themselves to remember to use SWEPPE. At follow-up, they described the value of notifications and reminders, and they also wished for recurrent and more frequent reminders.

#### Burden

##### Time Aspects

Prospectively, both patients and employers raised concerns about the time aspect of using SWEPPE. They hoped that it would not be too time consuming; however, the time aspect was not mentioned during follow-up.

##### Effort in Relation to Energy

Some patients found SWEPPE difficult to use, as they were feeling ill and had a lot of pain. When mental health was poor, the energy to focus on SWEPPE and provide good answers was just not there: “To be honest it has not been helpful to me. Actually, it has nothing to do with the app, rather I have been feeling really bad and had a lot of pain most of the time which have made me barely be able to register and use it as much as you should. I have not had any energy at all” (Patient 9).

One of the identified concerns was that the rating had to be done often, every day, which could get tedious and feel like a compulsion. Another concern was remembering the strategies and rating the strategies, which required a lot of effort, especially when not feeling well. One of the patients experienced phone use as stressful in itself, much like social media. She proposed that SWEPPE be made available in a nondigital form that could be handled in a more relaxed manner.

##### Technical Issues

At follow-up, both patients and one of the employers described technical issues that made using the app difficult, such as the disappearance of ratings, slow reloading of the graphs, and crashing of the app.

#### Ethicality: Privacy

At the time of introduction, one apprehension was that SWEPPE could negatively affect the employer’s view of the patient as a trustful and good employee. However, this was not further discussed by the patients at follow-up. Rather, some employers described the boundary related to private information shared by their employees. Questions were raised regarding information about training and meals and the importance of SWEPPE not being a tool for employers to monitor their employees.

## Discussion

### Principal Findings

In this study, the acceptability of SWEPPE was described from a user perspective. Overall, both patients and employers described SWEPPE as a supportive tool for increasing knowledge and understanding; identifying goals, barriers, and strategies; and improving employer-employee collaboration. However, there was a great variation among the different participants and modules in SWEPPE regarding acceptability.

The results from the questionnaires on acceptability in this study were comparable with the results from the development study. The thorough user-centered agile development of SWEPPE resulted in an app that was perceived by the reference groups as helpful, safe, relevant, logical, and easy to use for many patients with chronic pain [25]. In this study, it seemed like the acceptability of SWEPPE was good among the patients who were interested in and had the capability and enough energy to use SWEPPE continually. According to Rabenbauer and Mevenkamp [35], self-efficacy plays a significant role in compliance with eHealth interventions, as it can empower patients to participate in healthy activities [35]. In addition, other studies have raised the importance of self-efficacy and empowerment for the outcomes of interventions for patients with chronic pain in relation to general functioning [36] as well as work specifically [20]. The reference group in the development study expressed that SWEPPE needs to be quick and easy [25], which is how some of the participants in this study described SWEPPE. However, the results from this study show that when pain intensity is high and mental energy is low, it can be difficult to apply SWEPPE in daily life. That is, when support is most needed, low self-efficacy and empowerment might make it more difficult to acquire support. One of the ways to increase the acceptability of SWEPPE would be to increase the tailoring of SWEPPE to the individual’s needs. According to the participants in this study, flexibility and precision were appreciated. That is, the participants wanted to choose the ratings and strategies such that they would address their specific needs. This desire to tailor SWEPPE to individual needs is in line with the findings of Ledel Solem et al [9], who found that personalization and tailoring facilitated the use of eHealth interventions in pain management, including the choice of daily registrations of health or work aspects to meet the specific needs and challenges. Moreover, identifying the patients who can best benefit from SWEPPE is important. At the same time, as SWEPPE can be self-administered, it is easy to use if helpful but easy to reject if perceived as unhelpful.

Approximately half of the ratings regarding acceptability were lower retrospectively than prospectively. The test period started at the end of IPRPs and lasted for 3 months. This period is often a difficult time for patients, as the support from IPRP professionals and peers is no longer present [16]. Support from employers and other stakeholders is needed to fill this gap and continue the process of rehabilitation and RTW [37]. Internet-based self-management programs for chronic pain may be used to reduce the risk of end-of-rehabilitation-program crash [10]. The timing of SWEPPE after IPRP was experienced as good by both patients and employers. Knowledge and strategies from IPRPs can be used to identify relevant goals and understand graphs so as to monitor strategies and daily activities. The lower ratings retrospectively suggest the continuing need for support after IPRPs. For some patients, digital support such as SWEPPE can meet this need, but for others, there is a need for more professional support. However, the experience of a positive connection to IPRPs motivates further testing of SWEPPE for this group while broadening the testing for other groups.

The coach function in SWEPPE was used by 2 (18%) of the 11 patients, 1 time each. There was a low median rating of the coach function in the follow-up. This can be seen as a low acceptability of this function and questions its value in SWEPPE. However, the 2 patients who used the coach described substantial positive experiences, as the answers provided by the coach were helpful. The reasons for using or not using different modules in SWEPPE were not asked in the follow-up. Further development of the coach function is needed and has been initiated in another study.

The 4 employers who participated in the follow-up had employees who registered a weekly checkup for at least 7 weeks. Therefore, they were well informed about their employees’ goals and the weekly progress reported by SWEPPE. This may have been a motivator for participation in the follow-up and a basis for their answers in the interviews, which were substantially positive. One of the strengths of SWEPPE is that it starts with the patients’ and employees’ engagement, as it is their tool for self-management as well as for collaboration with their employers. However, when the relationship between employers and employees does not have a solid ground, it may be difficult for employees to share information and engage their employers. Conversely, without the employee’s engagement, it is not possible for the employer to take advantage of SWEPPE in developing their supporting role. Research has shown the importance of strengthening the employer’s role in the RTW process [19,38,39]. In later years, a tool for dialogue between employers and employees, the Demand and Ability Protocol (DAP), was tested in the Swedish context for patients with chronic pain. Using DAP during IPRPs may provide clear and straightforward communication regarding demands at work and facilitate the relationship between employees and employers. In addition, DAP can strengthen the connection between rehabilitation and work while facilitating a feeling of support and safety when health care is involved in the dialogue [40,41]. The findings of this study on the acceptability of SWEPPE after IPRPs point to the need for strengthening the relationship between employers and employees earlier to improve the acceptability of SWEPPE after IPRPs. Today, IPRPs are rarely used as a workplace intervention (ie, stakeholder meetings and workplace visits) [42]. A combination of DAP during IPRPs to build a foundation for communication and collaboration and SWEPPE after IPRPs to uphold and further develop the communication and collaboration could help some patients with more extensive needs for improving communication and collaboration with their employers.

### Strengths, Limitations, and Future Directions

There are a growing number of eHealth applications for chronic pain self-management that show promising results concerning pain intensity and disability [2,6]. However, no application before SWEPPE has focused on RTW or the involvement of employers. A strength of this study is that both qualitative and quantitative data were used [27-29] to describe the acceptability of SWEPPE. Using different data sources is a type of triangulation, which further increases the trustworthiness of the study [32]. In addition, the results from the interviews and questionnaires showed the same pattern, that is, a variation among the participants and modules of SWEPPE. Recurrent discussions among the authors of this study during the analysis ensured the credibility of coding and categorization, which, in turn, increased the trustworthiness of the results.

The focus of this study was on the acceptability of the intervention. We did not evaluate the methodological aspects of the forthcoming randomized controlled trial [43] such as the recruitment process, randomization, or outcome measures, which are other important aspects to investigate [29]. It was prioritized to focus on the user acceptability of SWEPPE to ensure that it is worth moving on to more large-scale studies in the context of IPRPs. In addition, because SWEPPE was developed with a user-centered design, it was valuable to study its acceptability in a real context. SWEPPE adds to the field, and the results of this study motivate further research.

One of the limitations of this study was the small number of participants, especially employers. The patient interviews resulted in rich data, and experiences were repeated in the final interviews. Data from the 4 employer interviews included both strengths and weaknesses of SWEPPE related to most components of the TFA. However, more interviews could have provided richer data, especially from the employer’s perspective. Results from the questionnaires should not be generalized owing to the small number of participants. Rather, the questionnaire results should be seen as complementing the qualitative part, triangulating and increasing the trustworthiness of the results.

In this study, there were an uneven distribution of women and men and an overrepresentation of social and caring workplaces, and most patients were aged approximately 40 years. These limitations must be considered when interpreting the transferability of the results. Including different types of workplaces and younger and older participants would have provided a wider representation and strengthened the transferability of the results. However, the participants of this study had participated in 4 different IPRPs within both primary and specialist care. The patients’ characteristics represented those of patients within IPRPs, a great majority of whom are women and whose mean age is approximately 40 years [44], which can be seen as a strength, as SWEPPE was developed for this group of patients.

When studying a preexisting theoretical structure in a new context, deductive qualitative content analysis can be used [34]. TFA was used to sort and categorize the acceptability of SWEPPE as described by the participants. This made it possible to structure the experiences concerning acceptability without missing important aspects. One of the challenges of using a deductive approach is handling the leftover data [32,45]. Leftover data in this study would include data related to the aim but outside the framework of TFA. However, no important data that could not be included in the TFA framework were identified. Rather, one aspect of acceptability was not mentioned by the participants that is, opportunity costs. As the data collection was open and not guided by TFA, we did not specifically ask about each aspect of acceptability. However, this does not mean that there were no opportunity costs; it just means that the participants in this study did not mention them in the interviews.

### Conclusions

SWEPPE was developed for patients with chronic pain and their employers to be used as a support for improved RTW after IPRPs. The first test of SWEPPE in this group showed promising results regarding user acceptability. SWEPPE was perceived to be easy to handle and was described as supportive for increasing knowledge and understanding, as well as for improving goals, strategies, and employer-employee collaboration. However, the acceptability of SWEPPE varied among the patients and modules. High degrees of flexibility and precision were appreciated and could increase acceptability. Excessive pain and low energy could hinder the use of SWEPPE, which suggests that SWEPPE might also be tested to prevent sick leave among persons with chronic pain, although not those with complex pain. Further development and research are needed to refine the modules and functions and identify patients who can best benefit from SWEPPE.

## Abbreviations

DAP: Demand and Ability Protocol
IPRP: interdisciplinary pain rehabilitation program
Q1E: questionnaire 1 for employers
Q1P: questionnaire 1 for patients
Q2E: questionnaire 2 for employers
Q2P: questionnaire 2 for patients
RTW: return to work
SWEPPE: Sustainable Worker Digital Support for Persons with Chronic Pain and Their Employers
TFA: theoretical framework of acceptability
